# LncRNA-RMRP promotes carcinogenesis by acting as a miR-206 sponge and is used as a novel biomarker for gastric cancer

**DOI:** 10.18632/oncotarget.9336

**Published:** 2016-05-12

**Authors:** Yongfu Shao, Meng Ye, Qier Li, Weiliang Sun, Guoliang Ye, Xinjun Zhang, Yunben Yang, Bingxiu Xiao, Junming Guo

**Affiliations:** ^1^ Department of Biochemistry and Molecular Biology and Zhejiang Key Laboratory of Pathophysiology, Ningbo University School of Medicine, Ningbo, 315211, China; ^2^ Department of Gastroenterology, The Affiliated Hospital of Ningbo University School of Medicine, Ningbo, 315020, China; ^3^ Ningbo Yinzhou People's Hospital and The Affiliated Hospital, Ningbo University School of Medicine, Ningbo, 315040, China; ^4^ Current address: Department of Gastroenterology, The Affiliated Hospital of Ningbo University School of Medicine, Ningbo, 315020, China

**Keywords:** gene expression, miRNA sponge, cell cycle, biomarker, gastric cancer

## Abstract

Long noncoding RNAs (lncRNAs) play crucial roles in tumorigenesis. However, the mechanisms of most lncRNAs in cancers are largely unknown. Because the RNA component of mitochondrial RNA processing endoribonuclease (RMRP) is one of the dysregulated lncRNAs in gastric cancer, this study explored its molecular mechanisms in carcinogenesis. RMRP levels in 792 tissues, plasma and gastric juices from patients with various stages of gastric tumorigenesis were analyzed by quantitative reverse transcription-polymerase chain reaction. Overexpression and RNA interference were used to manipulate RMRP expression by RMRP expression vector and small interfering RNAs, respectively. Its mechanisms were evaluated by flow cytometry, real-time cell analysis, plate colony formation assays, and xenograft models. RMRP levels in tissue, plasma and gastric juices from patients with gastric cancer were significantly different from those from controls. Its levels were significantly associated with Borrmann type and metastasis. Plasma and gastric juice RMRP had higher sensitivity and specificity than commonly used markers (such as carcinoembryonic antigen and carbohydrate antigen 19–9). Knockdown of RMRP significantly inhibited cell proliferation *in vitro* and *in vivo*, whereas overexpression of RMRP promoted cell growth. Acting as a miR-206 sponge, RMRP modulated cell cycle by regulating Cyclin D2 expression. RMRP plays a crucial role in gastric cancer occurrence and can be used as a novel biomarker for gastric cancer.

## INTRODUCTION

Gastric cancer remains the fourth most prevalent type of malignant tumor and the second leading cause of global cancer-related deaths [1–3]. Due to the absence of desirable biomarkers for early detection, more than 80% of gastric cancers are diagnosed at an advanced stage [3]. The unclear pathophysiologic mechanisms of gastric cancer have limited its clinical treatment options [4]. Therefore, identifying the molecular characterizations of gastric cancer and searching for new biomarkers are the major focuses of current research on gastric cancer [4, 5].

Long non-coding RNAs (lncRNAs) have been shown to be involved in physiological and pathological processes [6, 7]. The RNA component of mitochondrial RNA processing endoribonuclease (RMRP), an lncRNA, was first discovered in cartilage-hair hypoplasia (CHH), an autosomal recessive inherited disease [8]. RMRP is primarily identified in the nucleus, nucleolus and mitochondria [8, 9]. It is highly expressed in a wide range of human tissues and is essential for development at early stages of embryogenesis [10]. In mitochondria, RMRP helps endonuclease to cleave mitochondrial RNA at a priming site of mitochondrial DNA replication [11]. While in nucleoli, RMRP carries out an essential function in the final step of 5.8S rRNA processing [12]. It also interacts with the telomerase-associated reverse transcriptase (TERT) catalytic subunit to form a complex and produces double-stranded RNAs (dsRNAs), which are then processed into small interfering RNA (siRNA) by Dicer [13]. Despite the above-mentioned knowledge, the roles of RMRP in pathological processes, especially in carcinogenesis, remain unknown.

An increasing number of dysregulated lncRNAs in gastric cancer have been discovered in recent years [14–17]. RMRP was first found to exhibit dysregulated expression in gastric cancer in our previous study (GEO No. GSE47850: http://www.ncbi.nlm.nih.gov/geo/query/acc.cgi?acc=GSE47850) [15]. In this study, to clarify the potential roles of RMRP in gastric cancer and its clinical values, we first detected RMRP levels in tissue, plasma and gastric juice from patients with various stages of gastric tumorigenesis. Then, the molecular mechanisms underlying RMRP in gastric tumorigenesis were investigated. Our data showed that RMRP plays an important role in gastric cancer occurrence and development, and may be a potential novel biomarker for screening and predicting the prognosis of gastric cancer.

## RESULTS

### RMRP is downregulated during gastric carcinogenesis

**Figure 1:**
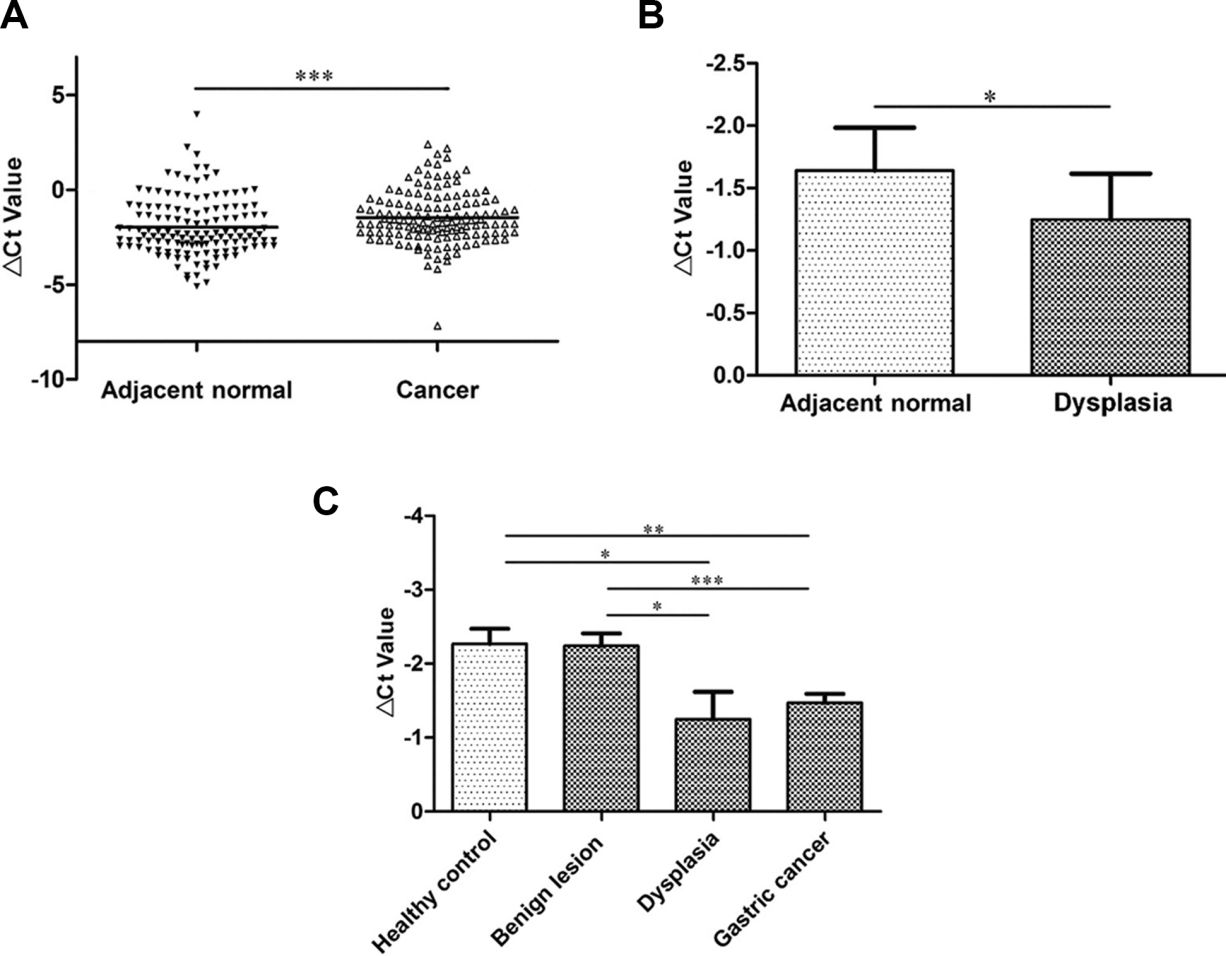
RMRP expression levels in gastric cancer tissues (**A**) RMRP expression levels were downregulated in 68.2% gastric cancer tissues compared with the paired adjacent non-tumorous tissues. (**B**) RMRP expression levels were significantly decreased in gastric dysplasia tissues compared with the healthy control group. *n* = 28. (**C**) RMRP expression levels in the tissues from various stages of gastric carcinogenesis. RMRP expression was only significantly decreased in gastric dysplasia (*n* = 28) and gastric cancer tissues (*n* = 132) compared with healthy controls (*n* = 37) and benign lesions (*n* = 34). RMRP expression levels were detected by qRT-PCR. Smaller Δ*C*_t_ value indicates higher expression. *P < 0.05, **P < 0.01, ***P < 0.001.

To verify the lncRNA microarray results [15], we expanded tissue sample numbers and found that its expression level was downregulated in 68.2% of gastric cancer tissues (*P* < 0.001; Figure 1A). We wondered whether RMRP levels have been changed during the course of gastric mucosal dysplasia. We further explored its levels in gastric dysplasia tissues. As shown in Figure 1B, the RMRP expression level was significantly downregulated in gastric dysplasia tissues (*P* < 0.05).

Finally, we investigated RMRP expression levels in the tissues from various stages of gastric carcinogenesis. RMRP expression was significantly downregulated in gastric dysplasia (*vs* healthy control or benign lesions, *P* < 0.05) and gastric cancer tissues (*vs* healthy control *P* < 0.01; *vs* benign lesions, *P* < 0.001; Figure 1C). The phenomenon of downregulation implies that RMRP has a strong correlation with gastric cancer occurrence.

### Relationship between tissue RMRP levels and clinicopathological factors of patients with gastric cancer

As shown in Supplementary Table 1, RMRP levels in gastric cancer tissue were significantly associated with Borrmann type (*P* = 0.002), tumor invasion (*P* = 0.037), lymphatic metastasis (*P* = 0.014), perineural invasion (*P* = 0.008), tissue carcinoembryonic antigen (CEA) levels (*P* < 0.001), and carbohydrate antigen 19–9 (CA19–9) levels (*P* = 0.003). These results imply that RMRP plays a role during gastric carcinogenesis.

### RMRP exists in human plasma and gastric juice

As body fluid is the main material used in the screening of cancers, we wondered whether RMRP might exist in human plasma and gastric juice. Thus, we sequenced the real-time quantitative reverse transcription-polymerase chain reaction (qRT-PCR) products of plasma and gastric juice RMRP. As expected, their sequences (Supplementary Figure 1) were completely consistent with the database (http://www.ncbi.nlm.nih.gov/nuccore/NR_003051.3).

The stability of body fluid lncRNAs is an important factor affecting the clinical application. As our data showed, there were no significant differences of RMRP levels among plasma of up to 8 cycles of freeze-thaw or under different time points (0, 2, 4, and 8 h) and incubation temperatures (4°C and 20°C) (Supplementary Figures 2 and 3; *P* > 0.05).

### Clinical diagnostic values of plasma RMRP

**Figure 2:**
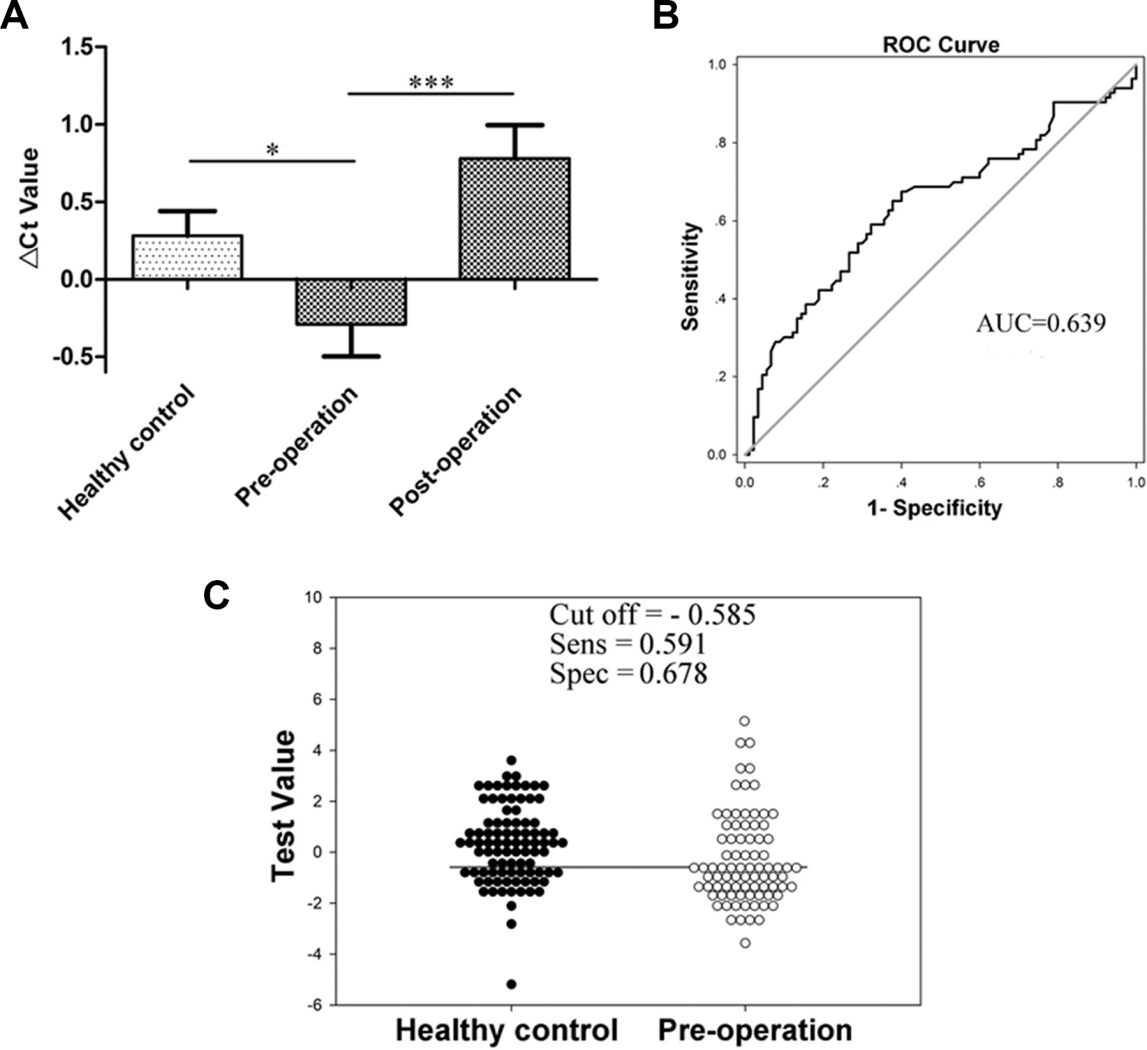
RMRP levels in plasma (**A**) RMRP levels in plasma from healthy control (*n* = 90), pre-operative (*n* = 83) and post-operative (*n* = 103) gastric cancer patients were detected by qRT-PCR. Smaller Δ*C*_t_ value indicates higher expression. *P < 0.05, ***P < 0.001. (**B**) The ROC curve. (**C**) The optimal cutoff value.

Blood RMRP levels were increased in the group of preoperative gastric cancer patients, but sharply declined in 72.3% (60/83) gastric cancer patients on day 15 after subtotal gastrectomy (Figure 2A). The area under the receiver operating characteristic (ROC) curve (AUC) was up to 0.639 (95% confidence interval [CI], 0.555–0.723; *P* < 0.001; Figure 2B). The sensitivity and specificity were 59.1% and 67.8%, respectively (Figure 2C).

The symbolic changes in plasma RMRP aroused our interest in exploring the potential correlations between plasma RMRP levels and clinicopathologic factors of patients with gastric cancer. The results showed that preoperative RMRP levels (Δ*C*_t_) were negatively correlated with tumor diameter (*P* = 0.031), stage (*P* = 0.038), invasion (*P* = 0.017) and tissue CEA expression (*P* = 0.032; Supplementary Table 2), whereas the individual relative changes (ΔΔ*C*_t_) of plasma RMRP after surgery had a significant and negative association with lymphatic metastasis (*P* = 0.040) and tissue CEA expression (*P* = 0.049; Supplementary Table 3). Additionally, the lower the preoperative or higher the postoperative RMRP level in plasma, the worse the pathologic result (Supplementary Tables 2 and 3). These results indicated that plasma RMRP has a potential as a biomarker for screening and predicting the prognosis of gastric cancer.

### Clinical diagnostic values of gastric juice RMRP

**Figure 3:**
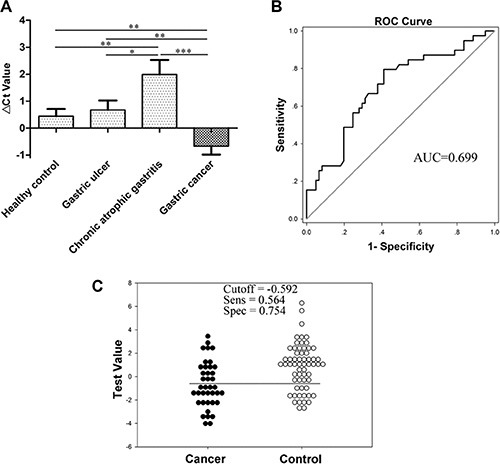
RMRP levels in gastric juice (**A**) RMRP levels in gastric juice from various stages of gastric carcinogenesis including healthy controls (*n* = 45), patients with gastric ulcers (*n* = 30), chronic atrophic gastritis (*n* = 16) and gastric cancer (*n* = 39) were detected by qRT-PCR. Smaller Δ*C*_t_ values indicate higher expression.*P < 0.05, **P < 0.01, ***P < 0.001. (**B**) The ROC curve. (**C**) The optimal cutoff value.

With a high specificity for the stomach, gastric juice has a significant advantage in reflecting upper digestive tract tumors [4]. We detected RMRP levels in human gastric juice from various stages of gastric carcinogenesis, including healthy controls, patients with gastric ulcers, chronic atrophic gastritis and gastric cancer. The results showed that RMRP levels significantly decreased in the group of chronic atrophic gastritis patients (*vs* healthy controls, *P* < 0.01; *vs* gastric ulcers, *P* < 0.05; *vs* gastric cancer, *P* < 0.001), but aberrantly increased in the gastric cancer group (*vs* healthy controls or gastric ulcers, *P* < 0.01; *vs* chronic atrophic gastritis, *P* < 0.001; Figure 3A). The AUC of gastric juice RMRP was up to 0.699 (95% CI, 0.593–0.805; *P* < 0.001; Figure 3B), which was higher than that of plasma RMRP. The sensitivity and specificity were 56.4% and 75.4%, respectively (Figure 3C). Compared with plasma RMRP, gastric juice RMRP has a higher diagnostic value, especially for specificity.

### Investigation of the source of RMRP in body fluid

Considering the above results, we were surprised to discover the following characteristics: First, compared with healthy group, RMRP levels were decreased in gastric cancer tissues (Figure 1A); however, they were aberrantly increased in plasma and gastric juice from patients with gastric cancer (Figures 2A and 3A). Second, plasma RMRP levels were aberrantly increased in preoperative gastric cancer patients, but sharply declined after subtotal gastrectomy, and were even lower than those in the healthy group (Figure 2A). Finally, gastric juice RMRP levels were significantly reduced in the group of chronic atrophic gastritis patients (Figure 3A), who exhibited less active secretion of gastric cells.

The above interesting phenomena greatly aroused our interest. We speculated that the main reason for these phenomena might the active secretion during gastric carcinogenesis. To verify this hypothesis, we cultured normal human gastric mucosa epithelial cells and gastric cancer cells in serum-free medium and performed qRT-PCR to measure RMRP levels in medium after 0, 8, 24, and 48 h of incubation. As expected, we found that RMRP levels in cell supernatant tend to increase with ongoing incubation (Supplementary Figure 4).

### Manipulation of RMRP expression level

To manipulate RMRP expression levels, siRNA-RMRP was transfected into normal gastric mucosa epithelial cells or gastric cancer cells. qRT-PCR analyses revealed that RMRP expression was effectively knocked down by si-RMRP (Supplementary Figure 5). A pcDNA3.1-RMRP vector was used to overexpress RMRP and the results confirmed its upregulated effects (Supplementary Figure 6).

### Effects of RMRP on cell proliferation

**Figure 4:**
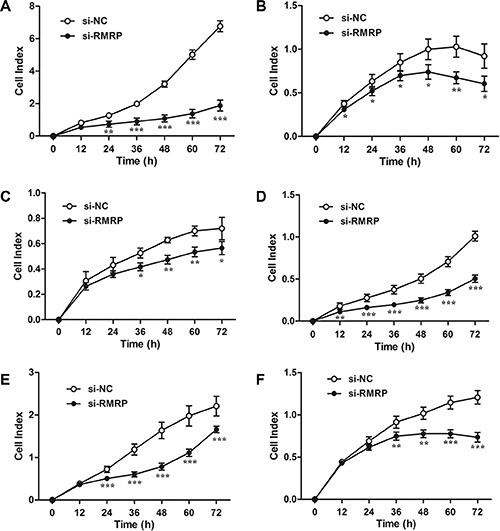
Results of cell proliferation after knockdown of RMRP Real-time cell analyzer (RTCA) reports showed that cell growth was significantly impaired in the human normal gastric epithelial cell line GES-1 (**A**) and the gastric cancer cell lines AGS (**B**), BGC-823 (**C**), HGC-27 (**D**), MGC-803 (**E**) and SGC-7901 (**F**) transfected with si-RMRP. Data are presented as the mean ± SD, *n* = 16. *P < 0.05, **P < 0.01, ***P < 0.001.

The relationships between tissue RMRP levels and clinicopathological factors of patients with gastric cancer suggested that RMRP might play a role during gastric carcinogenesis. Thus, we investigated the effects of RMRP on cell proliferation. The real-time cell analyzer (RTCA) revealed that cell growth was significantly impaired in a human normal gastric epithelial cell line (Figure 4A) and gastric cancer cell lines transfected with si-RMRP (Figure 4B–4F), whereas cell proliferation was increased in pcDNA3.1-RMRP-transfected cells (Supplementary Figure 7). Moreover, rescue experiments demonstrated that the overexpression of RMRP followed by knockdown of RMRP restored cell proliferation (Supplementary Figure 8). Similar results were observed by plate colony formation assays (Supplementary Figure 9).

### Downregulation of RMRP inhibits cell cycle proceed

**Figure 5:**
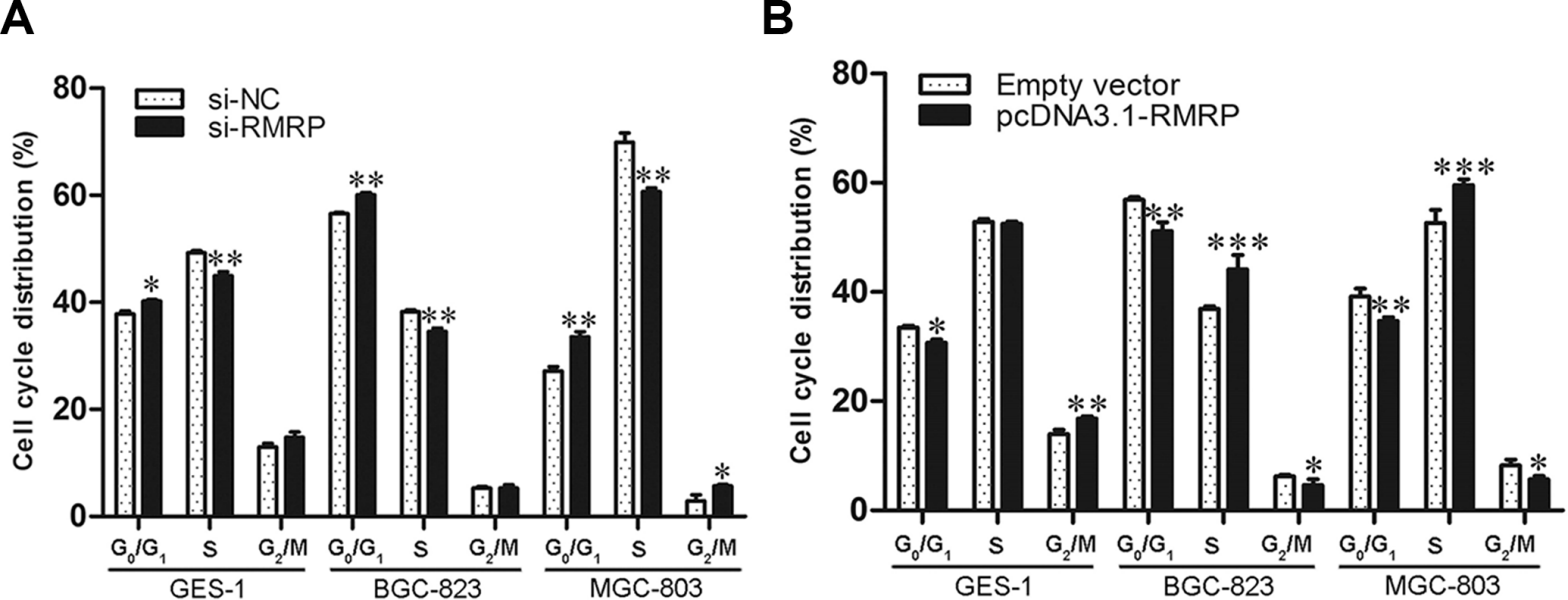
Cell cycle distribution after knockdown or overexpression of RMRP (**A**) The human normal gastric epithelial cell line GES-1 and the gastric cancer cell lines BGC-823 and MGC-803 transfected with si-RMRP represented significant G_0_/G_1_ arrest and S phage reduction compared with controls. (**B**) The overexpression of RMRP after pcDNA3.1-RMRP transfection arrested cells at the S phase. Data are presented as the mean ± SD, *n* = 3. *P < 0.05, **P < 0.01, ***P < 0.001.

Cell cycle and apoptosis contribute to cell proliferation. Therefore, we used flow cytometry to analyze cell cycle distributions. Normal human gastric mucosa epithelial cells and gastric cancer cells transfected with si-RMRP represented significant G_0_/G_1_ arrest and S phage reduction (Figure 5A). Inversely, overexpression of RMRP by pcDNA3.1-RMRP transfection increased the proportions of S phase (Figure 5B). Taken together, the regulatory effects of RMRP on cell proliferation in gastric cancer resulted from cell-cycle interruption.

### RMRP affects gastric cancer cell growth *in vivo*

**Figure 6:**
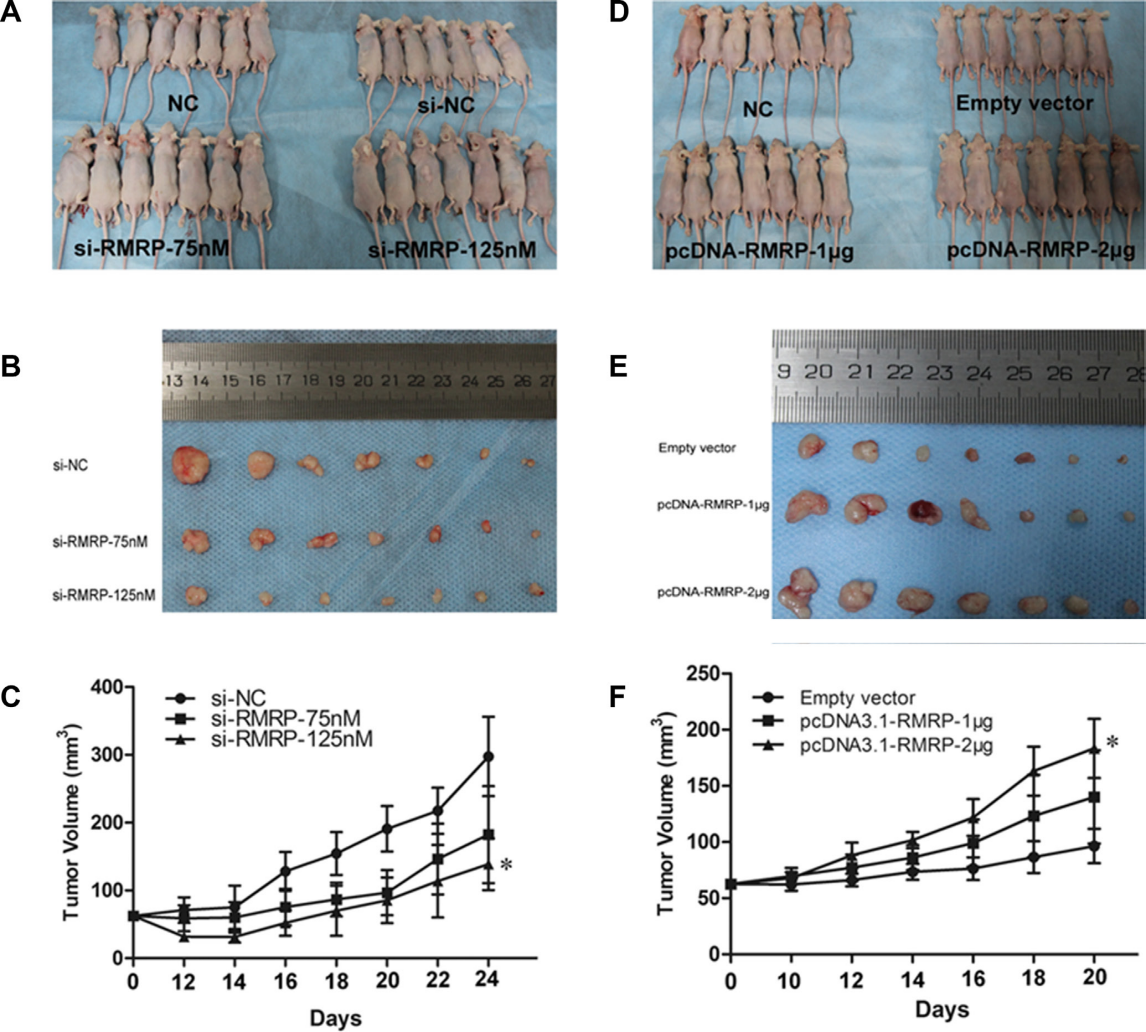
RMRP affects the growth and tumorigenicity of gastric cancer cells *in vivo* . (**A-C**) Knockdown of RMRP significantly inhibited tumor growth compared with the control group, as evidenced by the reductions in the dose-dependent tumor volume in the si-RMRP transfected group. (**D-F**) The growth of tumors transfected with pcDNA3.1-RMRP was obviously promoted in a dose-dependent manner. (A, D) Nude mice euthanized. (B, E) Gastric cancer tumor tissue from euthanized nude mice. (C, F) Tumor growth curve. *n* = 7. *P < 0.05.

To confirm whether RMRP affects gastric tumorigenesis *in vivo*, MGC-803 cells transfected with si-RMRP or si-NC were subcutaneously injected into mice. As shown in Figure 6A, 6B, and 6C, knockdown of RMRP significantly inhibited tumor growth in a dose-dependent manner. Contrarily, those transfected with pcDNA3.1-RMRP were obviously promoted in a dose-dependent manner (Figure 6D, 6E, and 6F).

Then, qRT-PCR analyses of RMRP levels in mice plasma were performed. The results showed that circulating RMRP levels in mice plasma were directly correlated with the RMRP levels in human gastric cancer cells (Supplementary Figure 10). Taken together, these results indicate that downregulated RMRP inhibited tumor growth *in vivo* and that body fluid RMRP was actively secreted from human gastric tissues.

### Cyclin D2 is the key downstream mediator of RMRP

**Figure 7:**
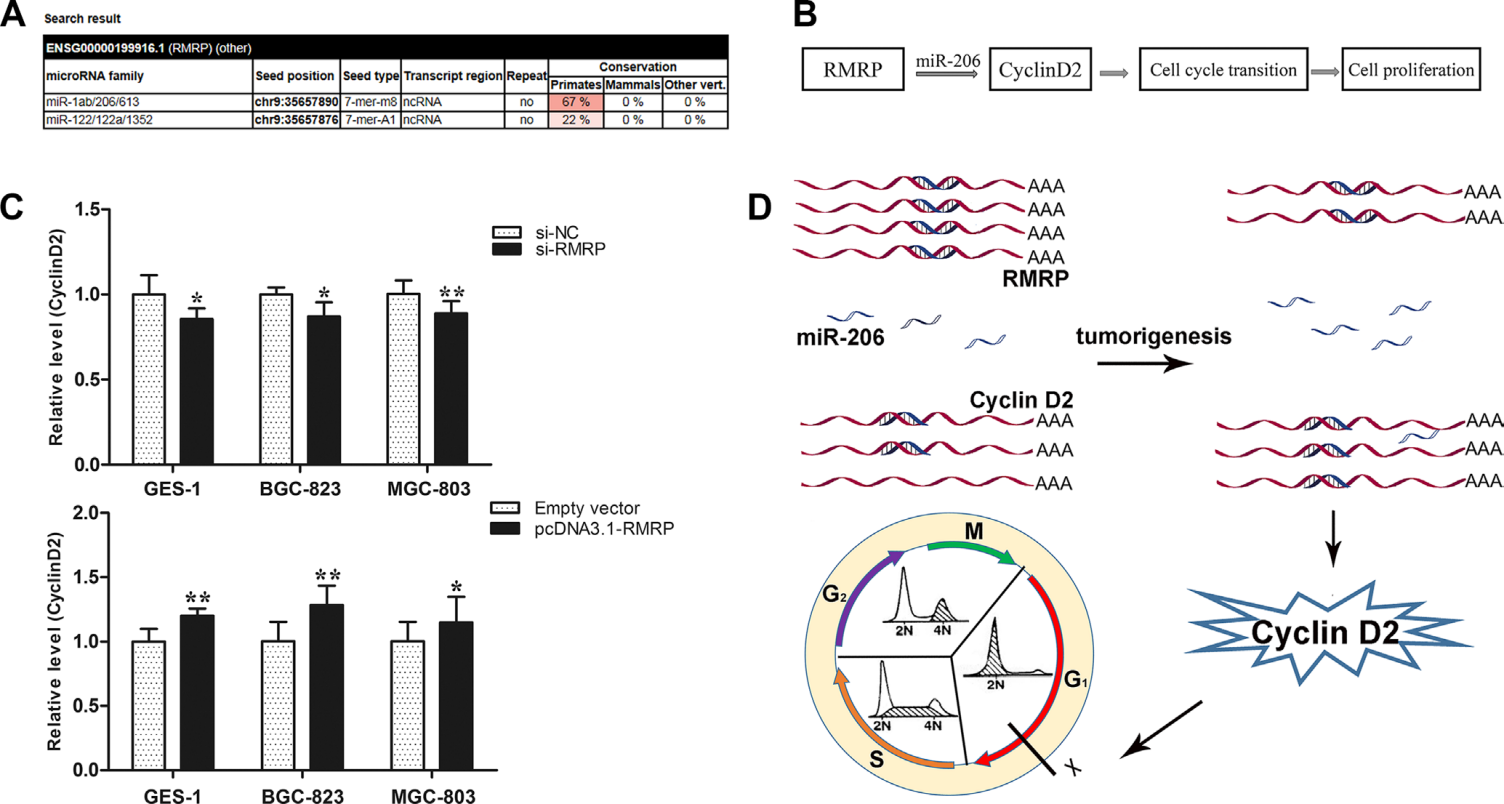
Cyclin D2 is the key downstream mediator of RMRP (**A**) A miRcode algorithm was used to predict RMRP-miRNA interaction. RMRP contains one seed sequence that can combine 6 miRNAs including miR-1a/b, miR-206, miR-613, miR-122, miR-122a and miR-1352. (**B**) A RMRP - miR-206 - Cyclin D2 network was constructed. (**C**) Cyclin D2 mRNA expression levels in human normal gastric epithelial cell line GES-1 and the gastric cancer cell lines BGC-823 and MGC-803 after knockdown or overexpression of RMRP. qRT-PCR was used to detect Cyclin D2 mRNA levels. Smaller Δ*C*_t_ value indicates higher expression. Data are presented as the mean ± SD, *n* = 3 *P < 0.05, **P < 0.01. (**D**) A model of RMRP - miR-206 - Cyclin D2 interaction. RMRP acts as a miR-206 sponge to modulate the cell cycle by regulating the expression level of the downstream target Cyclin D2.

Some lncRNAs, such as FER1L4 (fer-1-like family member 4, pseudogene), CCAT1 (colon cancer associated transcript 1) and SNAI1 (snail homolog 1), play important roles in the regulation of gene expression by acting as microRNA (miRNA) sponges [16, 18, 19]. We wondered whether RMRP acts as a miRNA sponge to regulate the cell cycle. By using the miRcode algorithm to predict RMRP-miRNA interactions, we found that RMRP contains one seed sequence that might combine with 6 miRNAs (Figure 7A). Then, we found that only miR-206 was reported to be closely associated with gastric cancer [20]. Moreover, miR-206 is a potential tumor suppressor with G_0_/G_1_ cell cycle arrest by targeting the Cyclin D2 [20]. Based on the results of miRcode algorithms and previous experiments, we constructed an RMRP - miR-206 - Cyclin D2 network (Figure 7B).

To verify whether this regulatory network exists, we detected Cyclin D2 mRNA levels in GES-1, BGC-823 and MGC-803 cells after knockdown or overexpression of RMRP. We found that knockdown of RMRP led to Cyclin D2 downregulation, whereas overexpression of RMRP led to Cyclin D2 upregulation (Figure 7C). Taken together, these results demonstrated that RMRP acts as a miR-206 sponge to modulate cell cycle through regulating the expression of Cyclin D2 (Figure 7D).

## DISCUSSION

lncRNAs play important roles in the occurrence and development of gastric cancer [4, 14, 21, 22]. In our previous research, we found that RMRP is one of dysregulated lncRNAs in the global lncRNA expression profile of gastric cancer [15]. The aim of the present study was to explore the molecular mechanisms of RMRP underlying gastric carcinogenesis and to investigate its diagnostic value.

Tumorigenesis is multistep processes [23]. The processes of gastric carcinogenesis are characterized by phenotypic multistep progression cascades [4, 23]. Dysplasia is a gastric precancerous lesion and is one key step for gastric carcinogenesis [24]. We first used qRT-PCR to detect RMRP expression levels between gastric cancer tissues and the paired non-tumorous tissues. We found that RMRP levels were downregulated in gastric cancer tissues (Figure 1A). Then, we investigated the expression pattern of RMRP among the healthy gastric mucosa, gastric ulcers, erosive gastritis, gastric dysplasia and gastric cancer tissues. The results showed that RMRP expression was significantly decreased in gastric dysplasia and gastric cancer tissues (Figure 1C). The phenomenon of tissue-specific downregulation indicated that RMRP has a strong correlation with gastric cancer.

Body fluid is the main material for clinical diagnosis. The stability of body fluid lncRNAs is an important factor affecting their clinical application. Our results confirmed the stability of body fluid RMRP (Supplementary Figures 2 and 3). This implies that the nature of body fluid RMRP meets the needs of clinical routine detection.

The sensitivity and specificity of plasma or gastric juice RMRP as biomarkers for gastric cancer screening are the focus of our research. Plasma collection is convenient, painless and acceptable, whereas gastric juice, with a high specificity for gastric organs, has a significant advantage in detecting upper digestive tract tumors. To assess the clinical value of plasma and gastric juice RMRP, we first analyzed the variation of plasma and gastric juice RMRP levels among various stages of gastric carcinogenesis. Our results showed that compared with healthy group, plasma RMRP levels aberrantly increased in the group of preoperative gastric cancer patients, but sharply declined after subtotal gastrectomy (Figure 2A). Gastric juice RMRP levels only significantly increased in the gastric cancer group (Figure 3A). These imply that plasma and gastric juice RMRP may be used as a biomarker for gastric cancer screening, and postoperative plasma has the potential to predict the prognosis of patients with gastric cancer. Our data indicated that the diagnostic value of gastric juice is higher than that of plasma RMRP (Figures 2C and 3C).

Body fluid exosomes are secreted by cells under both normal and pathological conditions [25–28]. Exosomes harbor diverse types of nucleic acids such as mRNA, miRNAs and lncRNAs, and actively participate in cell-to-cell communication by transferring cellular constituents from one cell to another [25, 27, 28]. Our previous study demonstrated that lncRNA-LINC00152 that exists stably in blood was protected by exosomes [29]. In our current study, we found that RMRP levels in cell supernatant tended to increase during incubation (Supplementary Figure 4). Moreover, animal experiments showed that circulating RMRP levels in mice plasma were directly correlated with RMRP levels in human gastric cancer cells (Supplementary Figure 10). Taken together, these results indicate that body fluid RMRP might be actively secreted by gastric tissues.

Age, tumor size, stage, invasion, lymphatic metastasis, perineural invasion, Borrmann type, and the expression of tissue CEA and CA19–9 are independent clinical prognostic factors in gastric cancer patients [30–34]. Borrmann type, tumor size, stage and invasion are valuable predictors for cancer metastasis and survival [30, 31, 34], whereas the presence of perineural invasion, tissue CEA and CA19–9, lymphatic metastasis, and age have been identified as independent prognostic factors for survival [31, 32, 33]. In our study, RMRP levels in gastric cancer tissues were associated with these clinicopathologic factors (Supplementary Table 1). Preoperative plasma RMRP levels were negatively correlated with tumor diameter, stage, invasion and tissue CEA expression (Supplementary Table 2), whereas the individual relative changes of plasma RMRP levels 2 weeks after surgery had a significant and negative association with lymphatic metastasis and tissue CEA expression (Supplementary Table 3). These results indicated that RMRP is also a potential biomarker to predict the prognosis of gastric cancer.

The balance between cell proliferation and apoptosis depends on the regulation of oncogenes, anti-oncogenes and growth factors [35, 36]. Cancers are diseases of inappropriate cell proliferation [37]. We discovered that the manipulation of RMRP expression levels in gastric cells has significant effects on cell proliferation *in vitro* and *in vivo*, and the effects of proliferation are associated with cell cycle (Figures 5 and 6). What is the underlying molecular mechanism of RMRP in regulating the cell cycle? Recent studies have demonstrated that some lncRNAs, such as FER1L4, CCAT1 and SNAI1, play important roles in the regulation of gene expression by acting as miRNA sponges [16–18]. We identified RMRP containing one seed sequence that might combine with 6 miRNAs (Figure 7A). Then, we confirmed that among these miRNAs, only miR-206 has been reported to be closely associated with gastric cancer. The RMRP - miR-206 - Cyclin D2 regulatory network was constructed (Figure 7B). Finally, we verified that knockdown of RMRP led to Cyclin D2 downregulation, whereas overexpression of RMRP led to Cyclin D2 upregulation (Figure 7C). Taken together, these results demonstrated that RMRP acts as a miR-206 sponge and modulates the cell cycle by regulating the expression of Cyclin D2 (Figure 7D).

Previous studies have shown that RMRP forms a complex with TERT, which exhibits RNA-dependent RNA polymerase activity and produces dsRNAs that can be processed into siRNAs by Dicer [13]. Recent research reported that nuclear RMRP acts as a reservoir for the production of a class of small RNAs [8]. Among the above genes, there are 9 genes related to apoptosis, 46 genes related to the cell cycle and 78 genes related to cancer [8]. This information implies that RMRP is most likely to act as a reservoir for the production of some small RNAs to regulate the cell cycle in gastric cancer. Moreover, research on CHH also found that the transcription start site of RMRP is highly conserved, and mutations in its promoter can drastically affect the rate of gene transcription [38].

In conclusion, the studied *in vivo* and *in vitro* mechanisms showed that lncRNA-RMRP plays a crucial role in the occurrence and progression of gastric cancer; and RMRP may be a potential biomarker for screening and predicting the prognosis of gastric cancer.

## MATERIALS AND METHODS

### Specimens

Specimens were obtained from three centers for gastroenterology, the Affiliated Hospital of Ningbo University School of Medicine, the First Hospital of Ningbo, and Yinzhou People's Hospital, between 2011 and 2013. Approximately 132 paired gastric cancer tissues and non-tumorous tissues (5 cm away from tumor) were collected from surgical patients. The 37 healthy gastric mucosa, 16 gastric ulcer, 18 erosive gastritis, and 28 paired gastric dysplasia tissues were obtained from biopsy specimens. All specimens were immediately preserved in RNA fixer (Bioteke, Beijing, China) at −80°C until use.

Peripheral venous blood was obtained from 90 healthy volunteers, 83 preoperative and 98 postoperative (2 weeks) gastric cancer patients after a 12-h overnight fast. Blood was collected in 9 ml ethylenediaminetetraacetic acid (EDTA) anticoagulation tubes (Kangjian, Taizhou, China). All plasma were separated into a 2 ml RNase-free centrifuge tubes (Axygen, Union, CA) and then stored at −80°C until use.

Gastric juice samples were obtained from 45 healthy volunteers, 30 gastric ulcer patients, 16 chronic atrophic gastritis patients, and 39 gastric cancer patients following previously described protocol [4].

Tumors were staged according to the tumor-node-metastasis (TNM) staging system of the International Union Against Cancer (5th ed). Histological grade was assessed following the National Comprehensive Cancer Network (NCCN) clinical practice guidelines for oncology (V.1.2011). No patient received local or systemic treatment before the upper gastrointestinal endoscopy examination or surgical excision. A double-blind study design was used. This study was approved by the Human Research Ethics Committee of Ningbo University, China (IRB No. 20120303). Written informed consent was obtained from all participants.

### RNA extraction and qRT-PCR detection

Tissue RNA and gastric juice/plasma RNA were extracted using TRIzol and TRIzol LS reagents (Ambion, Carlsbad, CA), respectively, following the manufacturer's instructions. The A260/A280 ratio and 1% agarose gel electrophoresis were used to assess the quality of RNA (Supplementary Figure 11). Then, total RNA was reverse transcribed to cDNA by a GoScript Reverse Transcription (RT) System (Promega, Madison, WI) and qRT-PCR analyses were performed with the GoTaq qPCR Master Mix (Promega) on an Mx3005P Real-Time PCR System (Stratagene, La Jolla, CA). The PCR primers for RMRP, glyceraldehyde-3-phosphate dehydrogenase (GAPDH) and Cyclin D2 were as follows: RMRP, 5′-ACTCCAAAGTCCGCCAAGA-3′ and 5′-TGC GTAACTAGAGGGAGCTGAC-3′; GAPDH, 5′-ACCC ACTCCTCCACCTTTGAC-3′ and 5′-TGTTGCTGTAG CCAAATTCGTT-3′; Cyclin D2, 5′-TGCTGTCTGCAT GTTCCTGGCCTC-3′ and 5′-ATCTTAGCCAGCAGCT CAGTCAGG-3′. Their relative expression levels were calculated using the 2^−ΔΔCt^ method with GAPDH as the control [4]. Lower Δ*C*_t_ values indicate higher expression. All data are expressed as the mean ± standard deviation (SD) of at least 3 independent experiments.

### Cloning and sequencing

The qRT-PCR products of plasma and gastric juice RMRP were purified using the UNIQ-10 PCR Product Purification Kit and cloned into the pUCm-T vector, and then sequencing was performed following the manufacturer's instructions (Sangon Biotech, Shanghai, China).

### Detection of CEA and CA 19–9 levels

The paraffin tissue sections were first incubated in primary anti-CEA or anti-carbohydrate antigen 19–9 (CA 19–9) antibody and then in broad-spectrum second antibody K5007 (DAKO, Glostrup, Denmark). After that, samples were incubated in diaminobenzidine (DAKO) for color development. The standard for the determination of results was in accordance with the 2010 American Society of Clinical Oncology (ASCO)/College of American Pathologists (CAP) guidelines.

An Elecsys 2010 instrument (Roche Diagnostics, Basel, Switzerland) was used to measure serum CEA and CA 19–9 levels with the cutoff values of 5 ng/ml and 35 U/ml, respectively.

Gastric juice CEA levels were measured using an enzyme-linked immunosorbent assay kit (KBH Diagnosis, Shanghai, China) with a SpectraMax M5 Microplate Reader (Molecular Devices Inc., Sunnyvale, CA). The cutoff value was 10 ng/ml.

### Cell culture

The normal human gastric mucosa epithelial cell line GES-1 and the gastric cancer cell lines AGS, BGC-823, HGC-27, MGC-803 and SGC-7901 were purchased from the Shanghai Institute of Biochemistry and Cell Biology, Chinese Academy of Sciences (Shanghai, China). Cells were cultured in RPMI-1640 Medium (Life Technologies, Grand Island, NY) supplemented with 10% fetal bovine serum (Life Technologies) in a humidified atmosphere at 37°C with 5% CO_2_.

### Plasmid construct and siRNA synthesis

To construct pcDNA3.1-RMRP expression vector, the entire sequence of human RMRP gene (NR_003051.3, 277bp) was synthesized and subcloned into a pcDNA3.1 (+) vector (GenePharma, Shanghai, China) (Supplementary Figure 12). The chemically modified siRNA oligo (2′ OMe) was synthesized by Shanghai GenePharma Co., Ltd. The sequences of three siRNA for the RMRP were 5′-CCUAGGCUACACACUGAGGACUTT-3′ (si-RMRP), 5′-UGCUGAAGGCCUAUAUCCUTT-3′(si-RMRP^#^), and 5′-GCCUGUAUCCUAGGCUACATT-3′(si-RMRP^*^). The sequence of negative control siRNA (si-NC) was 5′-UUCU CCGAACGUGUCACGUTT-3′.

### Transfection and cell proliferation assay

Normal human gastric mucosa epithelial cells and gastric cancer cells were transfected with pcDNA3.1-RMRP, empty vector, si-RMRP or si-NC using Lipofectamine 2000 reagent and Opti-MEM I Reduced Serum Medium (Life Technologies) in accordance with the manufacturer's instructions, respectively. Cell proliferation was analyzed with a real-time cell analyzer (RTCA; ACEA Biosciences, San Diego, CA) as previously reported [16].

### Flow cytometric analysis

Normal human gastric mucosa epithelial cells and gastric cancer cells were transfected with plasmid or siRNA. After 36 h of incubation, apoptosis was quantified using the fluorescein isothiocyanate (FITC)-Annexin V Apoptosis Detection Kit I (BD Biosciences, Sparks, MD). Cell cycle distributions were quantified using PI/RNase Staining Buffer (BD Biosciences) with a FACSCalibur flow cytometer (BD Biosciences). Experiments were performed in triplicate.

### Plate colony formation assay

After transfection for 24 h, cells were trypsinized to single cell suspensions and then seeded into 6-well plate. Two weeks later, the colonies were fixed with 4% paraformaldehyde (Bogoo, Shanghai, China) for 15 min and then stained with 0.1% crystal violet staining solution (Solarbio, Beijing, China). Experiments were performed in triplicate.

### Xenograft model experiment

Male BALB/c nude mice aged 6 weeks were purchased from Slac Laboratory Animal Center (Shanghai, China) and maintained under specific pathogen free (SPF) condition in the animal care facility at Ningbo University. A total of 3 × 10^6^ MGC-803 cells transfected with si-RMRP, pcDNA3.1-RMRP or control were suspended in 0.2 ml Matrigel Matrix (BD Biosciences) and then subcutaneously injected into the flanks of each mouse. The length (L, cm) and width (W, cm) of tumors were measured every 2 days starting the 10th day after inoculation. Tumor volume was calculated using the formula V = W^2^ × L × 0.5. Four weeks later, the mice were euthanized, and their blood was collected. All procedures were monitored in accordance with the ethical standards and the care of animal and licensing guidelines, issued by the administrative government, under the protocol approved by the Committee on Animal Welfare of Ningbo University.

### Statistical analysis

All statistical analyses were performed with Statistical Product and Service Solutions (SPSS) 20.0 software (SPSS, Chicago, IL). SigmaPlot12.3 (Systat Software, San Jose, CA) and GraphPad Prism 5.0 (GraphPad Software, La Jolla, CA) software were used to draw graphs. Student's *t*-test, one-way analysis of variance (ANOVA) and the rank-sum test were flexibly used according to actual conditions. *P* < 0.05 was regarded as statistically significant.

## SUPPLEMENTARY MATERIALS FIGURES AND TABLES


