# Supplementary material for: LncRNA-RMRP promotes carcinogenesis by acting as a miR-206 sponge and is used as a novel biomarker for gastric cancer
Source: Oncotarget. 2016 May 12;7(25):37812–24. doi: 10.18632/oncotarget.9336 (PMC5122351; doi:10.18632/oncotarget.9336)
Supplement: Supplementary file 1 [file oncotarget-07-37812-s001.pdf]

LncRNA-RMRP promotes carcinogenesis by acting as a miR-206 sponge and is used as a novel biomarker for gastric cancer

Supplementary Materials

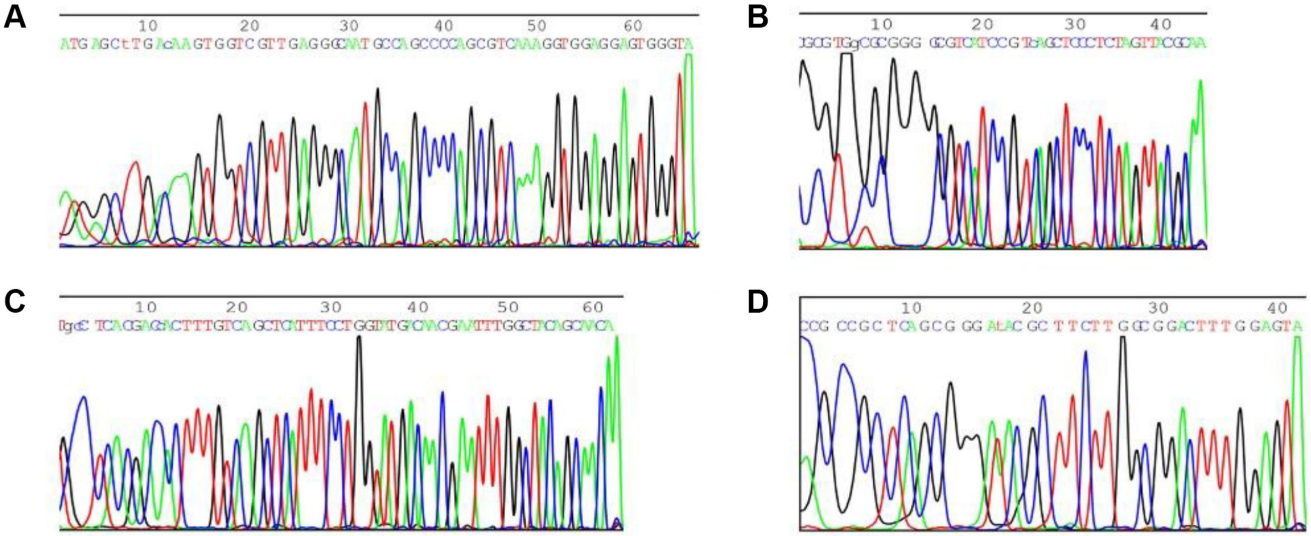

Supplementary Figure S1: DNA sequencing results of RMRP and glyceraldehyde-3-phosphate dehydrogenase (GAPDH). The qRT-PCR products of plasma and gastric juice RMRP and GAPDH were sequenced. (**A**) Plasma GAPDH. (**B**) Plasma RMRP. (**C**) Gastric juice GAPDH. (**D**) Gastric RMRP.

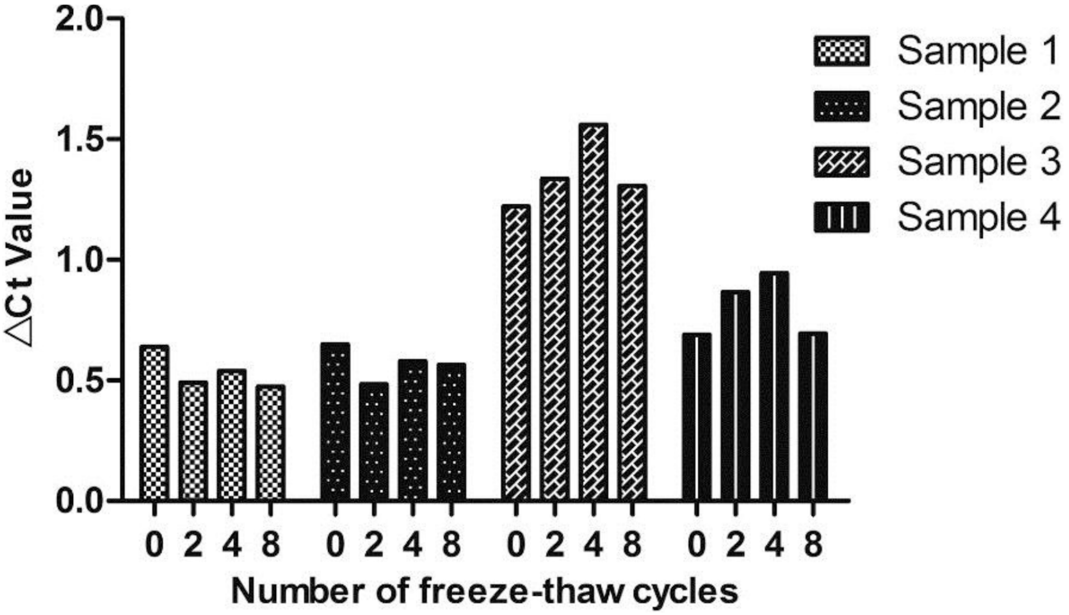

Supplementary Figure S2: Results of freeze-thaw experiments confirmed the stability of body fluid RMRP. Four blood samples were randomly selected and then equally divided to four parts. After 0, 2, 4, and 8 cycles of freeze-thaw, the RMRP levels were detected by qRT-PCR. Three independent experiments were performed. $P > 0.05$.

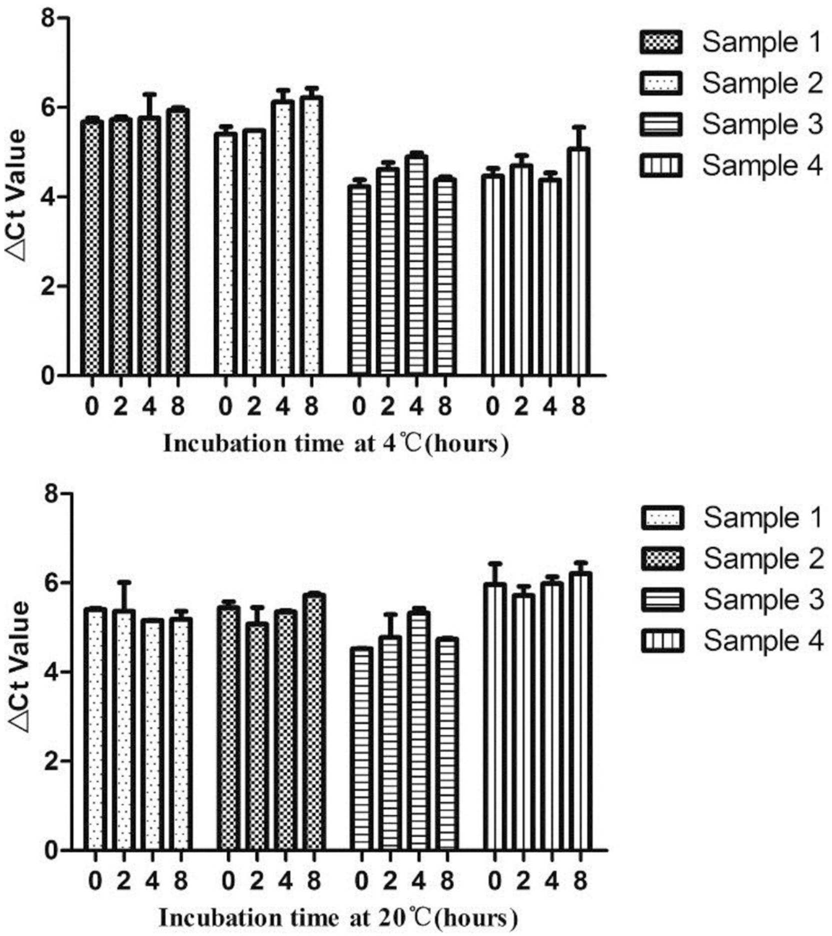

Supplementary Figure S3: Results of incubation experiments under different times and temperatures confirmed the stability of body fluid RMRP. Four blood samples were randomly selected and then equally divided to eight parts. Four of them were put at 4°C. Others were stored at 20°C. After 0, 2, 4, and 8 hour incubation, the RMRP levels were detected by qRT-PCR. Three independent experiments were performed. $P > 0.05$.

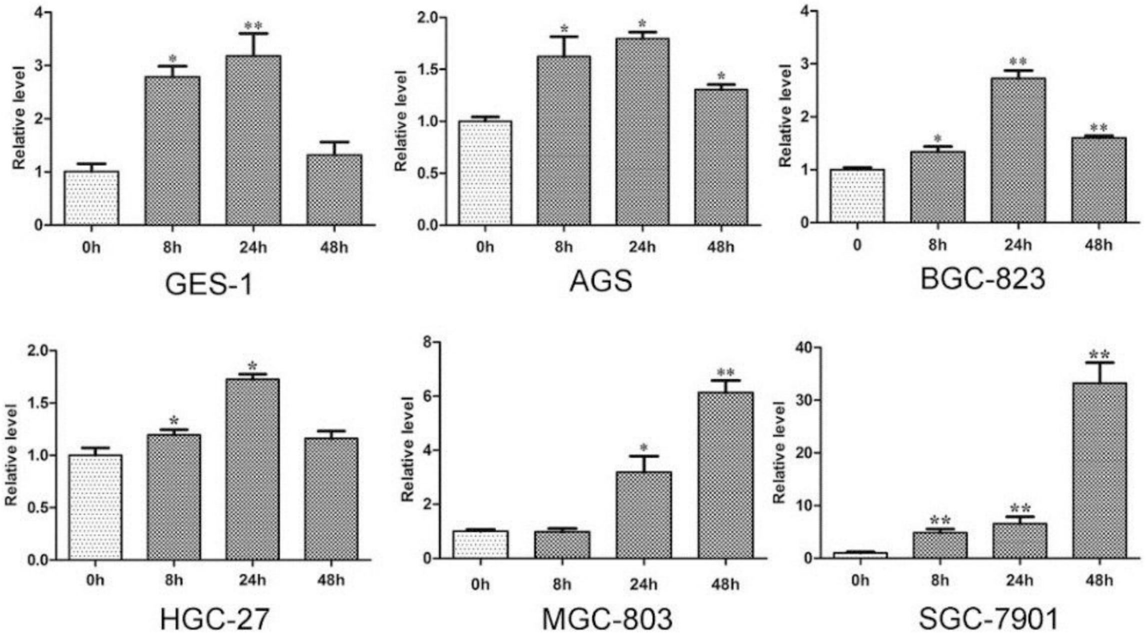

Supplementary Figure S4: Gastric cell culture experiments explored the source of RMRP in body fluid. Normal human gastric mucosa epithelial cell line GES-1 and gastric cancer cell lines AGS, BGC-823, HGC-27, MGC-803 and SGC-7901 were cultured in serum free medium. qRT-PCR was used to detect RMRP levels in medium after 0, 8, 24, and 48 h incubation. Three independent experiments were performed. $^{*}P < 0.05$, $^{**}P < 0.01$.

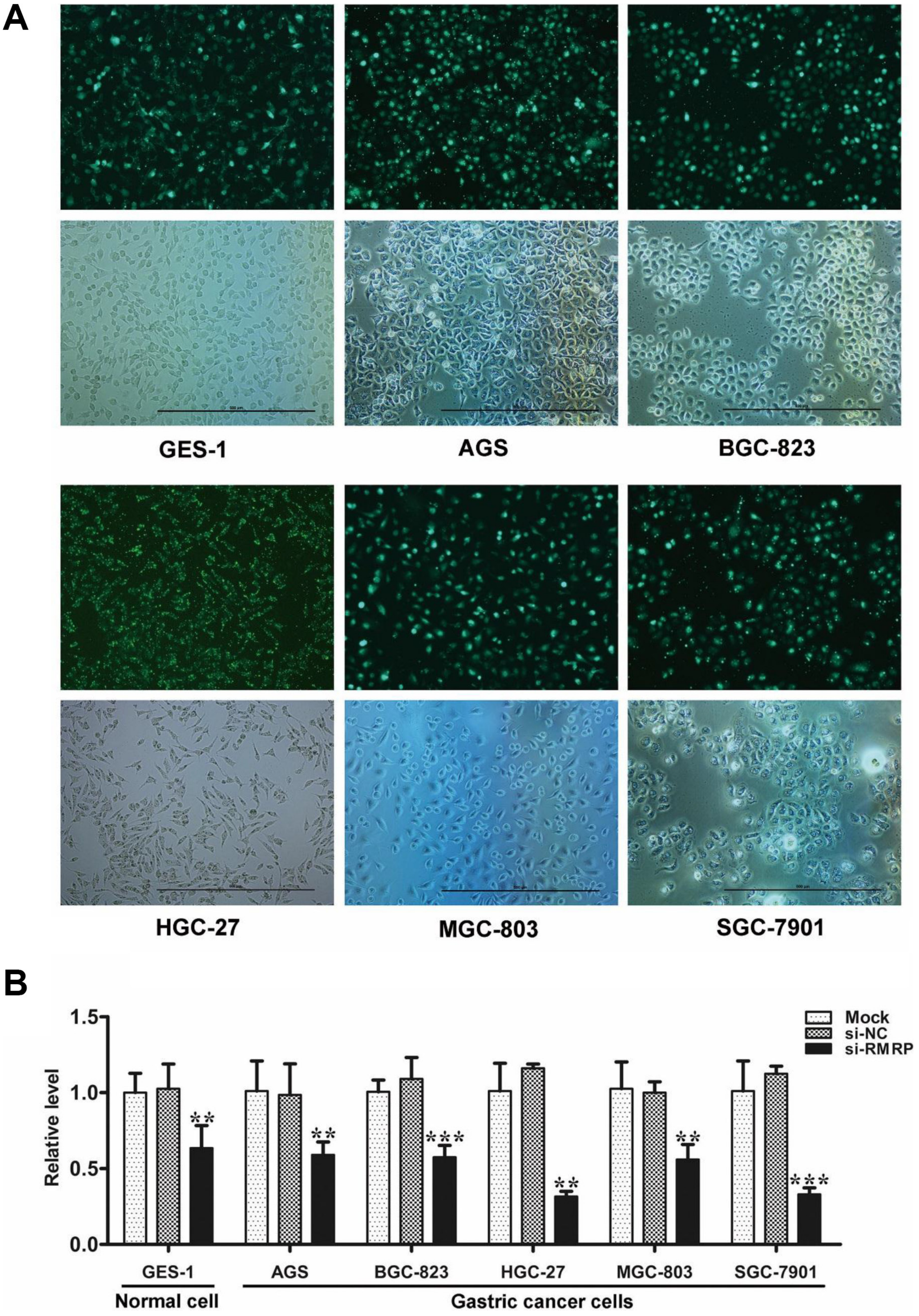

Supplementary Figure S5: Knockdown RMRP expression in gastric cells. (**A**) si-RMRP was transfected into normal human gastric mucosa epithelial cell line GES-1 and gastric cancer cell lines AGS, BGC-823, HGC-27, MGC-803 and SGC-7901. Up panel, fluorescence microscopic image; down panel, light microscopic image. Scale bars, 500 μm. (**B**) qRT-PCR was used to detect RMRP levels. Data are presented the as mean ± SD, $n = 3$. *$P < 0.05$, **$P < 0.01$, ***$P < 0.001$.

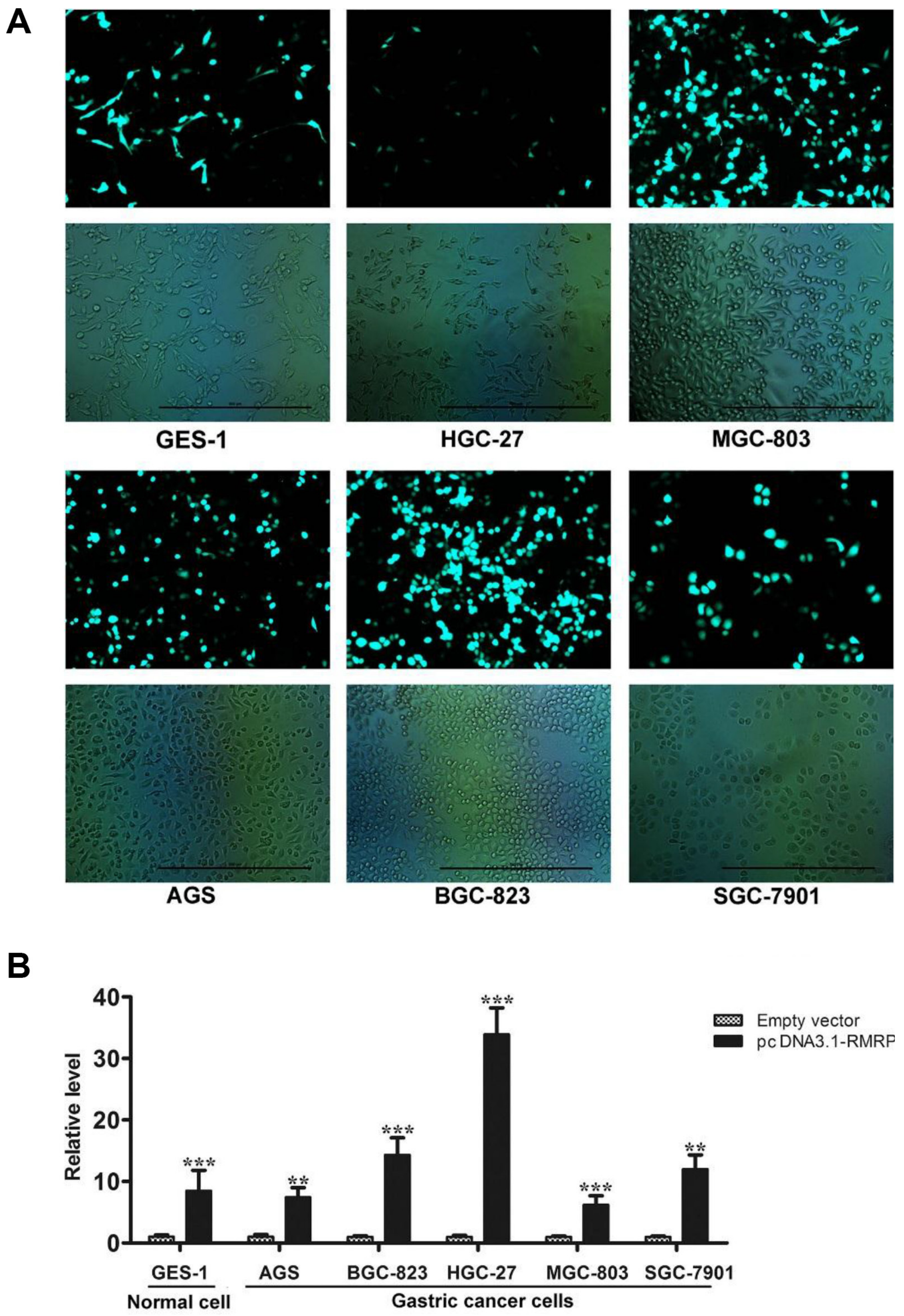

Supplementary Figure S6: Overexpression RMRP in in gastric cells. (**A**) pcDNA3.1-RMRP vector was transfected into normal human gastric mucosa epithelial cell line GES-1 and gastric cancer cell lines HGC-27, MGC-803, AGS, BGC-823 and SGC-7901. Up panel, fluorescence microscopic image; down panel, light microscopic image. Scale bars, 500 μm. (**B**) RMRP expression level was significantly increased in pcDNA3.1-RMRP-transfected groups compared with the empty vector control. Data are presented as the mean ± SD, $n = 3$. $^{**}P < 0.01$, $^{***}P < 0.001$.

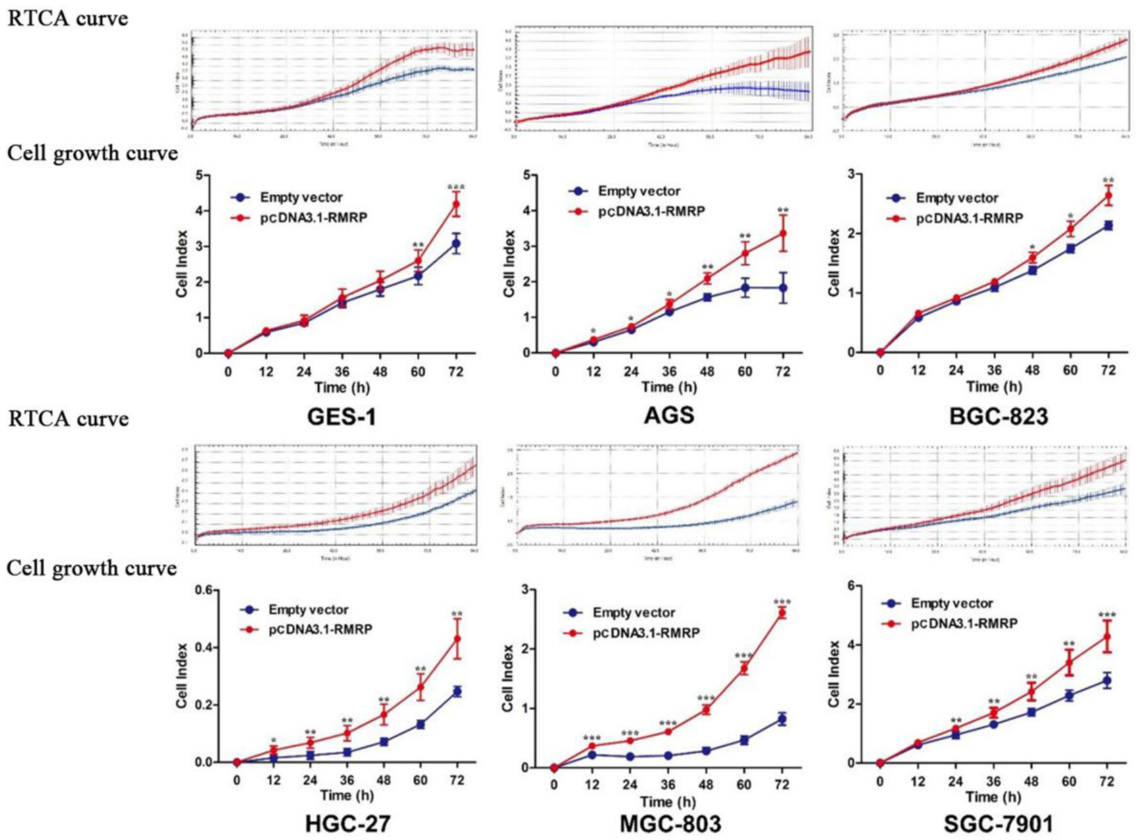

Supplementary Figure S7: Cell proliferation of normal human gastric mucosa epithelial cell line GES-1 and gastric cancer cell lines AGS, BGC-823, HGC-27, MGC-803 and SGC-7901 was increased in pcDNA3.1-RMRP transfected groups compared with respective controls. Data are presented as the mean ± SD, $n = 16$. *$P < 0.05$, **$P < 0.01$, ***$P < 0.001$.

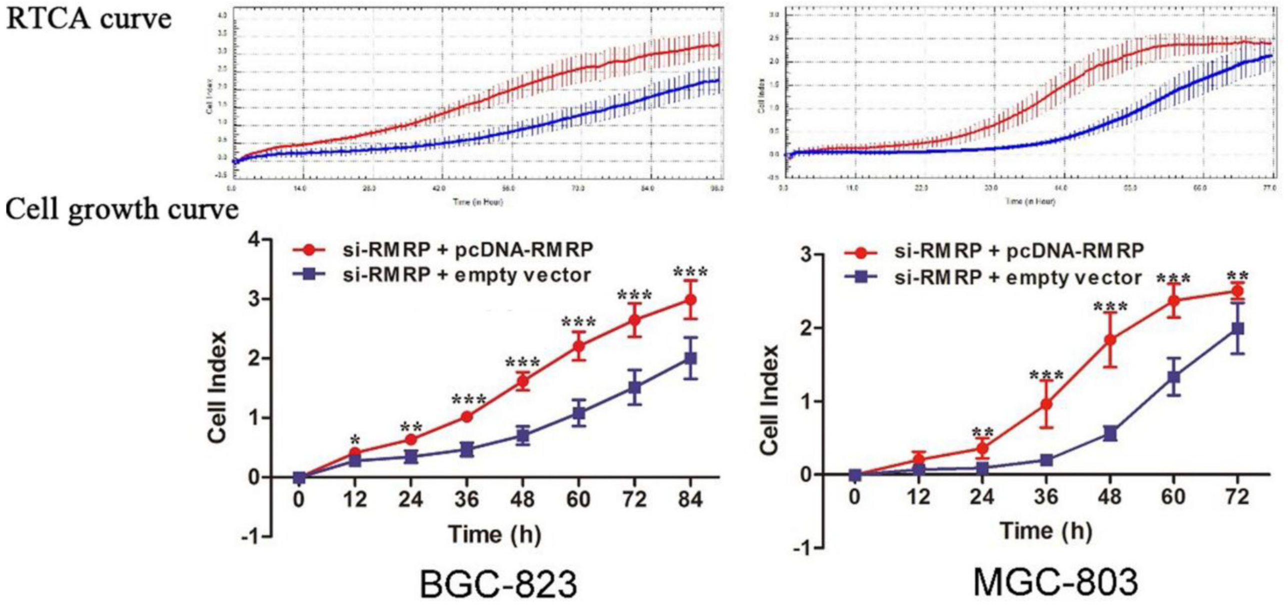

Supplementary Figure S8: Rescue experiments of cell proliferation about RMRP in BGC-823 and MGC-803. Rescue experiments demonstrated that overexpression of RMRP following by knockdown RMRP restored cell proliferation. Data are presented as the mean ± SD, $n = 16$. *$P < 0.05$, **$P < 0.01$, ***$P < 0.001$.

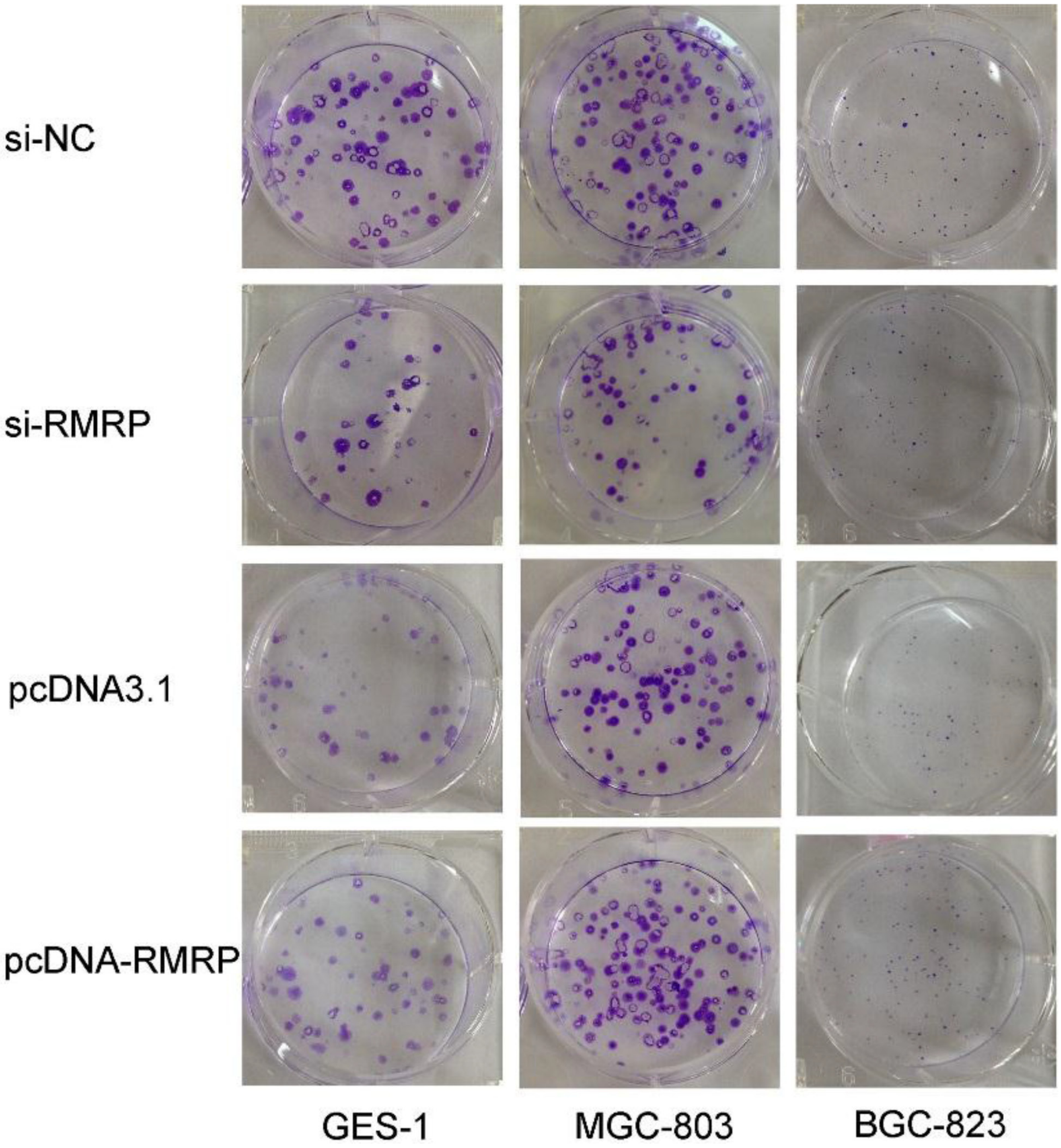

Supplementary Figure S9: Plate colony formation assays. Normal human gastric mucosa epithelial cell line GES-1 and gastric cancer cell lines MGC-803 and BGC-823 were transfected with si-negative control (si-NC), si-RMRP, pcDNA3.1 or pcDNA-RMRP. Two weeks later, the colonies were fixed with 4% paraformaldehyde and then stained by crystal violet staining solution. A representative results are showed.

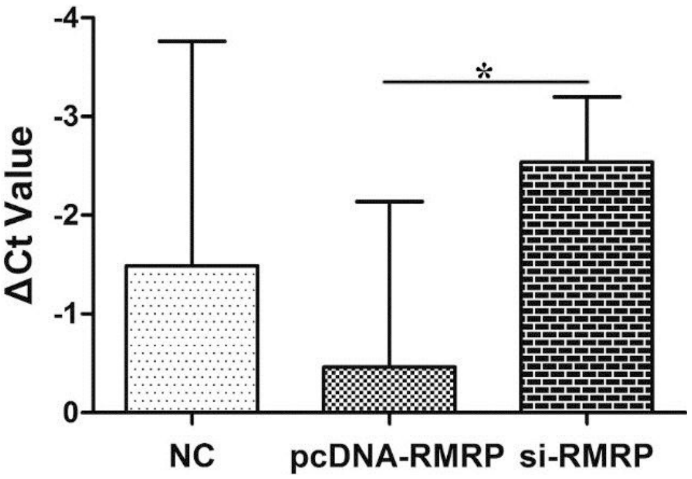

Supplementary Figure S10: RMRP levels in xenograft mice plasma. Male BALB/c nude mice were subcutaneously injected with Matrigel (negative control, NC) or MGC-803 cells transfected with pcDNA-RMRP or si-RMRP. Four weeks later, mice were euthanized and their blood was collected. qRT-PCR was used to detect RMRP levels. Smaller ΔC_t values indicate higher expression. Data are presented as the mean ± SD, $n = 14$. *$P < 0.05$.

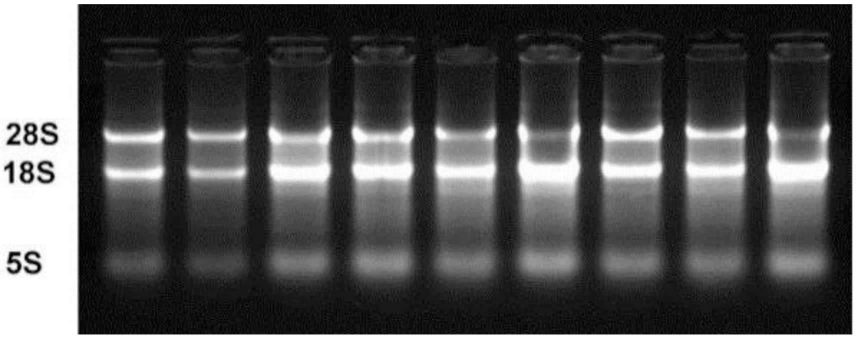

Supplementary Figure S11: Agarose gel electrophoresis of total RNA.

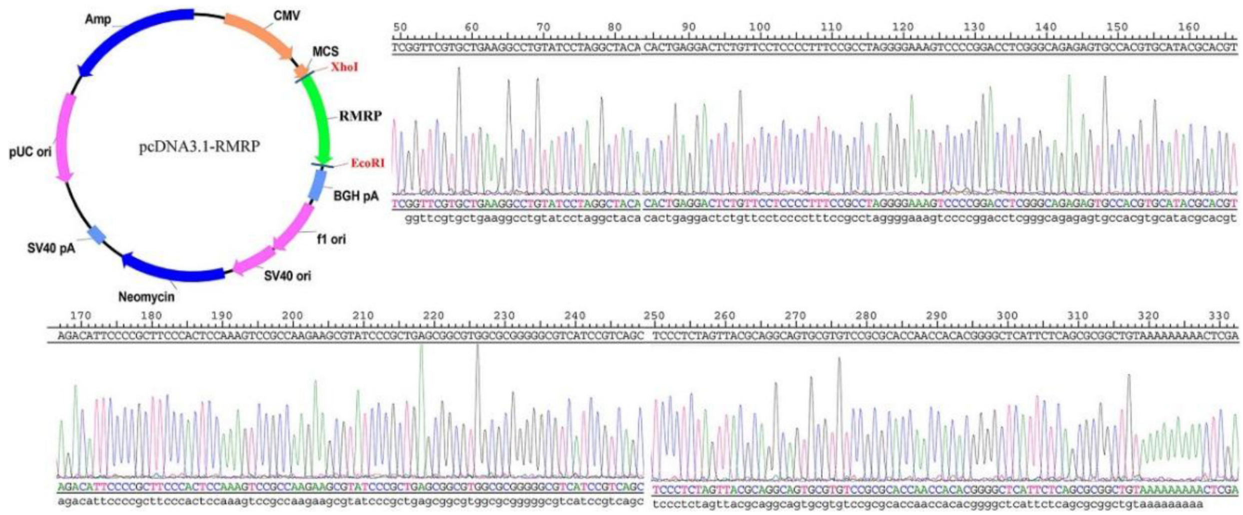

Supplementary Figure S12: pcDNA3.1-RMRP expression vector construct. RMRP was synthesized and subcloned into pcDNA3.1 (+) vector with incorporate external E*coRI* and X*hoI* sites. DNA sequencing was performed to confirm RMRP sequence.

Supplementary Table S1: Relationship of RMRP expression levels (ΔC_t) in cancer tissues with clinicopathological factors of patients with gastric cancer

Characteristics	No. of case (%)	Mean ± SD	*P* value
Age (y)			
≥ 60	88 (67)	–1.446 ± 1.263	0.947
< 60	44 (33)	–1.462 ± 1.436	
Gender			
Male	91 (69)	–1.482 ± 1.316	0.695
Female	41 (31)	–1.384 ± 1.335	
Tumor location			
Sinuses ventriculi	66 (50)	–1.285 ± 1.406	0.275
Cardia	19 (14)	–1.562 ± 1.075	
Corpora ventriculi	31 (23)	–1.823 ± 1.304	
Others	16 (13)	–1.289 ± 1.166	
Diameter (cm)			
≥ 5	65 (49)	–1.546 ± 1.230	0.421
< 5	67 (51)	–1.360 ± 1.400	
Differentiation			
Well	12 (9)	–1.748 ± 1.233	0.682
Moderate	65 (49)	–1.386 ± 1.229	
Poor	55 (42)	–1.465 ± 1.444	
Pathologic stage			
Early	31 (23)	–1.243 ± 1.359	0.316
Advanced	101 (77)	–1.515 ± 1.305	
Borrmann type			
I and II	20 (20)	–0.738 ± 1.288	0.002
III and IV	81 (80)	–1.707 ± 1.243	
Pathologic diagnosis			
Signet ring cell cancer	18 (14)	–1.231 ± 1.825	0.575
Adenocarcinoma	114 (86)	–1.486 ± 1.226	
Invasion			
T_1 and T_2	48 (36)	–1.168 ± 1.401	0.037
T_3 and T_4	84 (64)	–1.654 ± 1.186	
Lymphatic metastasis			
N_0	54 (41)	–1.115 ± 1.247	0.014
$N_{1–3}$	78 (59)	–1.684 ± 1.323	
Distal metastasis			
M_0	17 (13)	–1.792 ± 1.344	0.255
M_1	115 (87)	–1.401 ± 1.312	
Venous invasion			
Absent	69 (52)	–1.292 ± 1.298	0.146
Present	63 (48)	–1.626 ± 1.327	
Perineural invasion			
Absent	64 (48)	–1.142 ± 1.373	0.008
Present	68 (52)	–1.743 ± 1.202	
CEA (Tissue)			
Positive	100 (76)	–1.739 ± 1.114	< .001
Negative	32 (24)	–0.552 ± 1.507	

CA19-9 (Tissue)			
Positive	69 (52)	–1.778 ± 1.204	0.003
Negative	63 (48)	–1.094 ± 1.352	

Note: Smaller ΔC_t values indicate higher expression.

Supplementary Table S2: Relationship of RMRP levels (ΔC_t) in plasma before surgery with clinicopathological factors of patients with gastric cancer

Characteristics	No. of case (%)	Mean ± SD	*P* value
Age (y)			
≥ 60	55 (66)	–0.096 ± 2.128	0.196
< 60	28 (34)	–0.668 ± 1.296	
Gender			
Male	58 (70)	–0.480 ± 1.642	0.163
Female	25 (30)	0.156 ± 2.375	
Tumor location			
Sinuses ventriculi	42 (51)	0.007 ± 2.182	0.359
Cardia	11 (13)	–0.399 ± 0.993	
Corpora ventriculi	17 (20)	–0.972 ± 1.310	
Others	13 (16)	–0.255 ± 2.076	
Diameter (cm)			
≥ 5	42 (51)	0.044 ± 2.133	0.031
< 5	41 (49)	–0.629 ± 1.582	
Differentiation			
Well	7 (8)	–0.363 ± 2.220	0.661
Moderate	39 (48)	–0.093 ± 2.136	
Poor	37 (44)	–0.491 ± 1.563	
Pathologic stage			
Early	21 (25)	–1.030 ± 1.215	0.038
Advanced	62 (75)	–0.038 ± 2.029	
Borrmann type			
I & II	15 (24)	0.289 ± 1.625	0.478
III & IV	47 (76)	–0.142 ± 2.147	
Pathologic diagnosis			
Signet ring cell cancer	13 (16)	–0.694 ± 1.349	0.406
Adenocarcinoma	70 (84)	–0.213 ± 1.984	
Invasion			
T_1 & T_2	29 (35)	–0.876 ± 1.254	0.017
T_3 & T_4	54 (65)	0.027 ± 2.113	
Lymphatic metastasis			
N_0	35 (42)	–0.559 ± 1.399	0.272
$N_{1–3}$	48 (58)	–0.092 ± 2.189	
Distal metastasis			
M_0	71 (86)	–0.294 ± 1.903	0.954
M_1	12 (14)	–0.259 ± 1.964	
Venous invasion			
Absent	48 (58)	–0.556 ± 1.889	0.134
Present	35 (42)	0.078 ± 1.866	

Perineural invasion			
Absent	41 (49)	–0.376 ± 2.015	0.682
Present	42 (51)	–0.204 ± 1.801	
CEA(Tissue)			
Positive	57 (69)	–0.589 ± 1.733	0.032
Negative	26 (31)	0.372 ± 2.110	
CA19–9(Tissue)			
Positive	34 (41)	–0.348 ± 2.392	0.815
Negative	49 (59)	–0.248 ± 1.493	

Note: Smaller ΔC_t value indicates higher RMRP level.

Supplementary Table S3: Relationship of RMRP level changes ($\Delta\Delta C_t$) in plasma after surgery with clinicopathological factors of patients with gastric cancer

Characteristics	No. of case (%)	Mean ± SD	*P* value
Age (y)			
≥ 60	55 (66)	–0.513 ± 2.803	0.080
< 60	28 (34)	–1.374 ± 1.619	
Gender			
Male	58 (70)	–0.588 ± 2.553	0.218
Female	25 (30)	–1.335 ± 2.297	
Tumor location			
Sinuses ventriculi	42 (51)	–0.791 ± 2.033	0.110
Cardia	11 (13)	0.211 ± 4.036	
Corpora ventriculi	17 (20)	–1.932 ± 1.786	
Others	13 (16)	–0.305 ± 2.516	
Diameter (cm)			
≥ 5	42 (51)	–0.876 ± 2.204	0.786
< 5	41 (49)	–0.726 ± 2.793	
Differentiation			
Well	7 (8)	–1.321 ± 3.123	0.762
Moderate	39 (48)	–0.888 ± 2.722	
Poor	37 (44)	–0.617 ± 2.142	
Pathologic stage			
Early	21 (25)	–1.598 ± 1.958	0.091
Advanced	62 (75)	–0.535 ± 2.607	
Borrmann type			
I & II	15 (24)	0.373 ± 3.885	0.268
III & IV	47 (76)	–0.825 ± 2.015	
Pathologic diagnosis			
Signet ring cell cancer	13 (16)	–1.450 ± 1.669	0.311
Adenocarcinoma	70 (84)	–0.684 ± 2.607	
Invasion			
T_1 & T_2	29 (35)	–1.080 ± 2.960	0.462
T_3 & T_4	54 (65)	–0.655 ± 2.215	

Lymphatic metastasis			
N_0	35 (42)	–1.387 ± 2.215	0.040
$N_{1–3}$	48 (58)	–0.244 ± 2.631	
Distal metastasis			
M_0	71 (86)	–0.787 ± 2.481	0.878
M_1	12 (14)	–0.912 ± 2.676	
Venous invasion			
Absent	48 (58)	–0.800 ± 2.485	0.989
Present	35 (42)	–0.808 ± 2.537	
Perineural invasion			
Absent	41 (49)	–1.044 ± 3.007	0.389
Present	42 (51)	–0.569 ± 1.864	
CEA(Tissue)			
Positive	57 (69)	–1.166 ± 2.106	0.049
Negative	26 (31)	–0.009 ± 3.076	
CA19–9(Tissue)			
Positive	34 (41)	–0.708 ± 3.030	0.778
Negative	49 (59)	–0.867 ± 2.092	
Note: Smaller $\Delta\Delta C_t$ value indicates larger change.			
